# Serum Osteoprotegerin and Carotid Intima-Media Thickness Are Related to High Arterial Stiffness in Heart Failure with Reduced Ejection Fraction

**DOI:** 10.3390/diagnostics11050764

**Published:** 2021-04-24

**Authors:** Lajos Fehérvári, Attila Frigy, Lóránd Kocsis, István Adorján Szabó, Timea Magdolna Szabo, Melinda Urkon, Zita Jakó, Előd Ernő Nagy

**Affiliations:** 1 Department of Cardiology, Clinical County Hospital Mures, 540103 Targu Mures, Romania; fehervari.lajos@yahoo.com (L.F.); attila.frigy@umfst.ro (A.F.); kocsisfl@gmail.com (L.K.); sz.istvan.adorjan@gmail.com (I.A.S.); szabotimeamagdolna@gmail.com (T.M.S.); 2 Department of Internal Medicine IV, George Emil Palade University of Medicine, Pharmacy, Sciences and Technology of Targu Mures, 540103 Targu Mures, Romania; 3 Department of Biochemistry and Environmental Chemistry, George Emil Palade University of Medicine, Pharmacy, Sciences and Technology of Targu Mures, 540142 Targu Mures, Romania; melinda.urkon@umfst.ro; 4 Laboratory of Medical Analysis, Emergency Clinical County Hospital Targu Mures, 540136 Targu Mures, Romania; jakozita@yahoo.com; 5 Laboratory of Medical Analysis, Clinical County Hospital Mures, 540394 Targu Mures, Romania

**Keywords:** osteoprotegerin, calcification biomarkers, arterial stiffness, pulse wave velocity, heart failure

## Abstract

Arterial stiffness (AS) is a complex vascular phenomenon with consequences for central hemodynamics and left-ventricular performance. Circulating biomarkers have been associated with AS; however, their value in heart failure is poorly characterized. Our aim was to evaluate the clinical and biomarker correlates of AS in the setting of heart failure with reduced ejection fraction (HFrEF). In 78 hospitalized, hemodynamically stable patients (20 women, 58 men, mean age 65.8 ± 1.41 years) with HFrEF, AS was measured using aortic pulse wave velocity (PWV). Serum OPG, RANKL, sclerostin, and DKK-1 were determined, and the relationships between the clinical variables, vascular-calcification-related biomarkers, and PWV were evaluated by correlation analysis and linear and logistic regression models. OPG and the OPG/RANKL ratio were significantly higher in the group of patients (*n* = 37, 47.4%) with increased PWV (>10 m/s). PWV was positively correlated with age, left-ventricular ejection fraction, and carotid intima-media thickness (cIMT), and negatively correlated with the glomerular filtration rate. OPG and cIMT were significantly associated with PWV in the logistic regression models when adjusted for hypertension, EF, and the presence of atherosclerotic manifestations. Elevated serum OPG, together with cIMT, were significantly related to increased AS in the setting of HFrEF.

## 1. Introduction

Despite the important progress made in its diagnosis, evaluation, and treatment, heart failure (HF) remains one of the most important recent clinical practice challenges since it is associated with high morbidity and mortality and frequent hospitalizations. The pathophysiological concepts and treatment directions in HF are complex and different in the two distinct clinical forms of the disease: HF with preserved ejection fraction (HFpEF) and HF with reduced ejection fraction (HFrEF) [1]. The latter represents the classical form, with systolic dysfunction of the left ventricle being responsible for cardiac decompensation [2].

Concerning the underlying regulatory pathways, a shift toward an inflammatory phenotype and neuroendocrine activation has been described, with elevated expressions of TNF-α, TNF-α-related apoptosis-inducing ligand (TRAIL), myeloperoxidase, C-reactive protein (CRP), and monocyte chemoattractant protein 1 (MCP-1) [1]. Numerous biomarkers of the disease have been proposed; these offer valuable information concerning the disease’s pathophysiology and, in some cases, also possess prognostic and therapeutic value. Among candidate molecules, markers of myocardial stress, injury, oxidative stress, mediators of matrix and cellular remodeling, inflammation, and neuro-hormonal factors were previously outlined [3].

The macrovascular function is a less frequently studied phenomenon in the setting of HF; however, it is a well-known fact that arterial stiffness (AS) has a consistent effect on central hemodynamics, affecting left-ventricular function by altering the ventriculo–arterial coupling. In the case of increased AS, the earlier arrival of the reflected pulse wave causes an increase in left-ventricular afterload, which is responsible for left-ventricular hypertrophy, diastolic dysfunction (impaired relaxation), left atrial enlargement, and subendocardial ischemia (induced by increased oxygen demand). Furthermore, coronary perfusion is decreased by the reflected pulse wave’s movement toward the systole [4,5,6].

High AS is associated with a significant increase in the number of incident HFs [7]. The increase in AS is an age-related phenomenon involving various mechanisms, such as the degradation of elastin, abnormal collagen deposition, smooth muscle cell (SMC) dysfunction, and osteogenic differentiation, along with media calcification. Certain conditions, such as smoking, diabetes, hypertension, obesity, and chronic kidney disease, could accelerate the process. The evaluation of AS has gained more interest in clinical practice in the last two decades due to the development of feasible devices that are capable of measuring parameters reflecting AS. Among these parameters, pulse wave velocity (PWV) is considered to be the most robust and relevant. The clinical relevance of PWV has been studied in many clinical conditions, e.g., it is strongly linked to coronary and extensive cerebral artery calcification [8].

The osteoprotegerin (OPG)–receptor activator of nuclear kappa B ligand (RANKL)–receptor activator of nuclear kappa B (RANK) triad is a common regulator of bone remodeling and atherosclerotic plaque development [9]. The RANKL–RANK signaling controls osteoclast differentiation in the bone and the osteoid metaplasia of the vascular wall; OPG functions as a decoy receptor for RANKL, decoupling it from RANK [10]. It seems that in the course of atherogenesis, both vascular SMCs and bone-marrow-derived stem cells are able to undergo osteoblast-like differentiation [11]. These cells and the secreted OPG could contribute to media-type calcification. High extracellular phosphate in renal failure, hypercalcemia, increased local inflammatory cytokines, and mineralization-competent microvesicles released by SMCs, along with oxidative stress, all contribute to this process.

Osteoblast-like differentiation is also largely controlled by the Wnt/β-catenin signal pathway. The canonical Wnt signaling system and its inhibitors are other potent modulators of the vessel wall architecture, integrity, and vascular cell phenotype. Suppressors, such as sclerostin, downregulate the tissue turnover but enhance the SMC-mediated calcium deposition in the media. In clinical studies, the serum Wnt inhibitor sclerostin was significantly increased in chronic kidney disease patients with intensive vascular calcification [12].

Our work aimed to evaluate the prevalence and the clinical and biomarker correlations of AS in subjects with HFrEF. To achieve these, we measured serum OPG, RANKL, sclerostin, and another Wnt signal inhibitor, namely, DKK-1, in a cohort of HFrEF patients with various comorbidities and set up regression models for the determinants of PWV as a significant indicator of AS. These biomarkers’ role and significance in the prediction of AS in HFrEF are not yet well established.

## 2. Materials and Methods

### 2.1. The Study Cohort

In a prospective study, we enrolled 78 patients (21 female—26.9%; 57 male—73.1%; mean age 65.80 ± 1.41 years) with HFrEF, who were hospitalized during 2017–2020 in the Cardiology Department of the Clinical County Hospital Mures, Targu Mures, Romania. The inclusion criteria were as follows: willingness to participate in the study, left-ventricular ejection fraction <45%, hemodynamic stability (regarding blood pressure, rhythm, heart rate, and congestive signs), and a sinus rhythm. The exclusion criteria were as follows: an acute decompensation phase (regarding clinical status and therapy) and technical difficulties in determining AS. HFrEF was diagnosed based on symptoms, clinical signs, and the presence of left-ventricular systolic dysfunction found via echocardiography. Each patient underwent systematic data collection regarding routine demographic, clinical, laboratory, and echocardiographic data. Ischemic etiology of HF was considered based on their myocardial infarction history and/or significant lesions via previous coronarography. Hypertension and diabetes were defined in accord with current guidelines [13,14]. The estimated glomerular filtration rate (eGFR) was calculated using the Modification of Diet in Renal Disease (MDRD) formula [15].

Data handling thoroughly respected the Declaration of Helsinki, the patients signed an informed consent regarding participation, and the study was approved (3865/01.03.2016) by the Ethical Committee of Clinical County Hospital Mures.

### 2.2. Measurement of PWV

AS was characterized and assessed using PWV measurements. For measuring aortic PWV, the Mobil-O-Graph NG^®^ device (IEM GmbH, Stolberg, Germany) was used, which is a validated and approved device that uses a cuff-based oscillometric, non-invasive method. The recorded brachial pressure data (single-point detection via the dominant upper arm) were processed using the ARCSolver algorithm (Austrian Institute of Technology, Vienna, Austria) using a transfer function. In this way, central (aortic) pressure curves were obtained and the PWV was calculated.

PWV values >10 m/s were considered pathognomonic for increased AS. PWV measurements were performed in a supine position, in a quiet environment, excluding smoking or a meal in the hour before the examination. In every patient, two measurements were made, and the mean of the two PWV values was used [16].

### 2.3. Cardiac and Carotid Echocardiography and the Ankle–Brachial Index Determination

The echocardiography was performed using a Philips Epiq7 ultrasound machine (Philips Ultrasound, Inc., Bothell, WA, USA) according to the current recommendations. Two-dimensional apical two- and four-chamber views were used for volumetric measurements. The left-ventricular ejection fraction (LVEF) was determined using the modified Simpson’s method [17]. For the evaluation of the macrovascular arterial involvement, the ankle–brachial index (ABI) using standard peripheral CW Doppler examination (5 MHz) and the carotid intima-media thickness (CIMT) using 2D vascular echography (7 MHz linear probe, Aloka Prosound Alpha 10 (Aloka Gmbh, Meerbusch, Germany) were also measured. The cut-off value of >0.9 mm was used for increased CIMT [13].

### 2.4. Laboratory Analysis

Blood samples were drawn after overnight fasting into vacutainer tubes with no additive, with K3 EDTA, or with 3.2% trisodium citrate (Becton-Dickinson Vacutainer Systems, Wokingham, Berkshire, UK). The tubes without additive and with citrate were centrifuged at 3000 rpm for 10 min to separate the serum and plasma. The K3 EDTA vacutainers were used for complete blood count analysis on a Cell-Dyn Ruby analyzer (Abbott Laboratories, Diagnostic Division, Abbott Park IL, USA). Fasting glucose, total- and HDL-cholesterol, serum triglycerides, creatinine, uric acid, serum iron, and C-reactive protein were measured with commercial biochemical kits on an Arhitect C4000 (Abbott Laboratories, Diagnostic Division, Abbott Park, IL, USA). Plasma fibrinogen was determined using a coagulometric method with Multifibren U reagent on Sysmex CA-1500 (Sysmex Corporation, Kobe, Japan). Serum NT-proBNP was measured using an electrochemiluminescent immunoassay on Elecsys 2010 (Roche Diagnostics International, Rotkreuz, Switzerland).

### 2.5. Biomarker Measurements 

Blood samples were centrifuged for 10 min at 3000 rpm/min; after that, the serum was separated in 1.5 mL Eppendorf tubes and frozen at −50 °C. Commercially available ELISA kits were applied to analyze the serum concentrations of OPG (R&D Systems, Minneapolis, MN, USA, Human Osteoprotegerin/TNFRSF11B Duoset ELISA, DY805), RANKL (R&D Systems, Minneapolis, MN, USA, Human TRANCE/RANKL/TNFSF11 Duoset ELISA, DY626), sclerostin (R&D Systems, Minneapolis, MN, USA, Human SOST/Sclerostin Duoset ELISA, DY1406), and DKK-1 (R&D Systems, Minneapolis, MN, USA, Human Dkk-1 Duoset ELISA, DY1906). Complementary reagents were from the Duoset ELISA Ancillary Reagent Kit 2, and reactions were read on a Personal Lab ELISA automated instrument (Adaltis, Milano, Italy). The intra-assay variabilities of these immunoassays were the following: 6.4% (OPG), 4.8% (RANKL), 5.5% (sclerostin), and 6.2% (DKK1).

### 2.6. Statistical Analysis

The data distribution characteristics were analyzed using the Kolmogorov–Smirnov and Shapiro–Wilk tests. For variables with a normal distribution, we performed paired *t*-tests and Pearson’s correlation analyses. For variables with an abnormal distribution, we applied nonparametric statistical tests: Mann–Whitney *U* test for between-group comparisons and Spearman rank correlation analysis. Categorical variables were analyzed for their absolute and relative frequency. We constructed multiple linear regression models to determine PWV as a continuous variable in the whole group and the normal PWV subgroup. Finally, we set up nonlinear logistic regression models for the prediction of the high PWV values. Data processing was performed using Microsoft Excel 2016 (Microsoft Corporation, Redmond, WA, USA) and GraphPad Prism 9.01 (GraphPad Software LLC., San Diego, CA, USA).

## 3. Results

### 3.1. Study Group Characteristics: Factors Related to a Higher PWV

**Figure 1:**
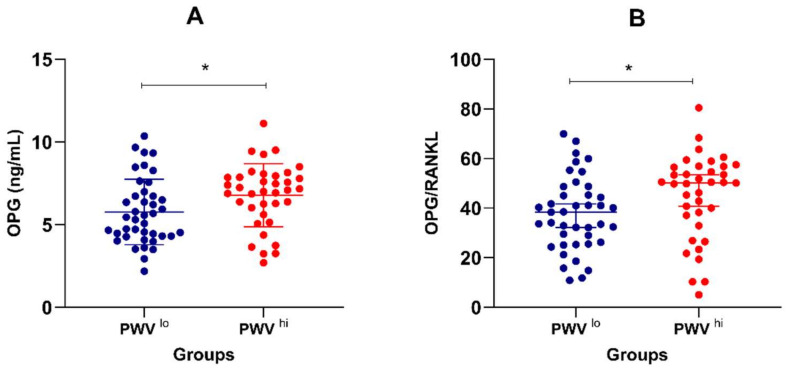
OPG concentrations (**A**) and OPG/RANKL ratios (**B**) in the two PWV subgroups. * *p* < 0.05.

Thirty-seven patients (47.4%) showed high PWV values (>10 m/s, PWV^hi^) according to the ESH-ESC 2018 guidelines, whereas in 41 cases, the PWV was in the normal range (PWV^lo^). In comparison, the PWV^hi^ group was older (*p* < 0.0001) and had higher glycemia (*p* = 0.038), higher serum OPG (*p* = 0.016), and a higher OPG/RANKL ratio (*p* = 0.029) (Figure 1A,B), as well as a higher intima-media thickness (*p* < 0.0001), a higher incidence of hypertension (*p* = 0.043), a higher left ventricular ejection fraction (*p* = 0.002), a lower left-ventricular end-diastolic diameter (*p* = 0.043), a lower glomerular filtration rate (*p* = 0.033), and a lower calcium-channel blockers usage (*p* = 0.048). There was no significant difference between the PWV of patients with and without a history of atrial fibrillation.

The prevalence of diabetes was similar in the two groups. The main clinical, functional, and laboratory parameters of the whole group and their comparisons are shown in Table 1.

### 3.2. Correlations of PWV, OPG, and Sclerostin

For the whole group of 78 patients, PWV was significantly correlated with age (*r* = 0.87, *p* < 0.0001), EF (*r* = 0.34, *p* = 0.002), left-ventricular end-diastolic diameter (*r* = −0.30, *p* = 0.006), GFR (*r* = −0.38, *p* = 0.0004), CRP (*r* = 0.26, *p* = 0.021), glycemia (*r* = 0.23, *p* = 0.039), OPG (*r* = 0.38, *p* = 0.0005), OPG/RANKL ratio (*r* = 0.37, *p* = 0.0009), sclerostin (*r* = 0.29, *p* = 0.009), and intima-media thickness (*r* = 0.55, *p* < 0.0001). If we classified the subgroups according to the PWV values, a significant correlation with serum HDL-cholesterol (*r* = 0.45, *p* = 0.003) in the PWV^lo^ (PWV ≤ 10 m/s) group, and an association with serum iron levels in the PWV^hi^ (PWV > 10 m/s) group (*r* = −0.33, *p* = 0.045) were found. Interestingly, OPG and the OPG/RANKL ratio were correlated with PWV only in the PWV^hi^ group (*r* = 0.33, *p* = 0.042 and *r* = 0.38, *p* = 0.018, respectively), in opposition to sclerostin, which showed a significant correlation (*r* = 0.37, *p* = 0.017) only in the PWV^lo^ subgroup.

In the whole group, we observed significant correlations of OPG with IMT (*r* = 0.22, *p* = 0.048) and GFR (*r* = −0.37, *p* < 0.001). Sclerostin also correlated with the two variables: IMT (*r* = 0.30, *p* = 0.007) and GFR (*r* = −0.25, *p* = 0.024).

The correlations for all continuous variables are presented in Table 2.

### 3.3. Correlations of PWV in the EF Subgroups

Thirteen (37.1%) patients with EF > 30% possessed a normal PWV, while 22 (62.9%) had high PVW values. In contrast, 28 (65.1%) cases with a very low ejection fraction (EF ≤ 30%) showed normal PVW and 15 (34.9%) had elevated values. The difference between the observed frequencies was significant (*p* = 0.022). The two EF subgroups also had some dichotomy in their correlations. In the group with EF ≤ 30%, PWV was significantly correlated with age (*r* = 0.88, *p* < 0.0001), left-ventricular end-diastolic diameter (*r* = −0.37, *p* = 0.014), glomerular filtration rate (*r* = −0.35, *p* = 0.019), and serum sclerostin (*r* = 0.38, *p* = 0.011). In the group with EF > 30%, positive correlations with age (*r* = 0.70, *p* < 0.0001) and blood glucose levels (*r* = 0.44, *p* < 0.007) and a negative correlation with GFR (*r* = −0.45, *p* = 0.006) were found. No correlation with sclerostin was observed *(r* = 0.22, *p* = 0.20); instead, OPG (*r* = 0.51, *p* = 0.0017) and the OPG/RANKL ratio (*r* = 0.52, *p* = 0.0014) were strongly associated with PWV. NT-proBNP was significantly higher in the very low ejection fraction group than in those with EF > 30%: 2789 (1268–4293) pg/mL vs. 1493 (549.1–2694) pg/mL, *p* = 0.002. There was a negative correlation between EF and serum NT-proBNP (*r* = −0.371, *p* = 0.0008), but we could not observe associations between PWV and serum NT-proBNP in the whole group nor in the PWV subgroups.

### 3.4. Predictors of PWV in the Multiple Linear Regression and Logistic Regression Models

We set up multiple linear regression models for the determinants of PWV as a continuous variable. Age was excluded from the models as the factor with the strongest correlation with PWV in these statistics, overlying other relationships. In a forward stepwise regression model including all cases, the most significant predictors were shown to be the intima-media thickness (multiple *R* = 0.50, *p* < 0.0001), gender (*R* = 0.58, *p* = 0.002), and the serum OPG level (*R* = 0.628, *p* = 0.010) when the model was adjusted for hypertension, left-ventricular end-diastolic diameter, serum sclerostin, and the level of physical exercise (Table 3A).

Since the correlates were different in the PWV^lo^ group, we performed a multiple linear regression restricted to this group. This model revealed intima-media thickness (multiple *R* = 0.675, *p* < 0.0001), serum sclerostin (*R* = 0.727, *p* = 0.020), and left ventricular end-diastolic diameter (*R* = 0.763, *p* = 0.035) as determinants of PWV when the model was adjusted for hypertension (Table 3B).

Nonlinear logistic regression models were constructed for the prediction of high PWV in the whole study group. We applied age-dependent cut-off values for serum NT-proBNP as follows: 450 pg/mL (<50 years), 900 pg/mL (50–75 years), and 1800 pg/mL (>75 years) [18]. In the first model, which was adjusted for ejection fraction and serum NT-proBNP, the odds ratio conferred by serum OPG in the upper tertile was 3.02 (1.13–8.07), with *p* = 0.032, while the odds ratio conferred by the intima-media thickness in the upper tertile was 3.75 (1.40–10.01), with *p* = 0.029 (Table 4, model 1).

In the second model, the odds of having OPG and IMT in the upper tertile vs. the lower tertile remained significant (*p* = 0.039 and *p* = 0.017) when the model was adjusted for hypertension and the presence of atherosclerotic manifestations (coronary disease, carotid stenosis, or peripheral arterial disease) (Table 4, model 2).

## 4. Discussion

Increased central AS, which is a distinctive sign of aging, is associated with cardiovascular diseases, including stroke, coronary heart disease, and HF. Prospective clinical studies highlighted that greater AS predicts a higher incidence of HF. Tsao et al. revealed in Framingham Study participants with no clinical HF that their carotid–femoral PWV (cfPWV) at baseline was associated with incident HF in a graded and continuous manner [7]. In the CRIC study that enrolled 2602 patients with 3.5 years of follow-up, cfPWV was found to be an independent predictor of incident hospitalized HF [19]. However, in other long-term studies, such as the Health ABC, the significant relationship between baseline cfPWV and the risk of HFrEF was abolished after adjustment for traditional cardiovascular risk factors [20]; moreover, PWV proved to be less elevated in HF patients than in age-matched individuals with cardiovascular risk factors and no HF [21].

Vascular calcification is a major determinant of AS [8]. One crucial underlying pathway of this process is the phenotype shift of vascular SMCs, which are characterized by an osteoblast-like differentiation and osteoid metaplasia that is associated with the local expression of OPG and the plasma OPG/RANKL ratio [22].

According to the accumulated evidence, OPG is an active mediator of atherosclerotic vascular alterations in the intima, promoting endothelial cell proliferation and angiogenesis, but defends against vascular SMC calcification, suppressing the effects of RANKL [23]. OPG circulates in the bloodstream either as a monomer or a disulfide-bond-forming dimer [23] and is released upon specific pro-inflammatory stimuli (IL-1, TNF-α) from the Weibel–Palade bodies of endothelial cells, together with the platelet aggregation promoter Von Willebrand factor [24]. In some circumstances, OPG behaves as a pro-inflammatory mediator and exerts pro-atherogenic activity by inhibiting TRAIL [25]. In animal experiments, it has been found to prevent calcification, as OPG−/− mice downregulate their pro-inflammatory mediators, even in ischemic conditions [26], and show spontaneous vascular calcium deposition [27]. However, clinical studies suggest a positive correlation between circulating OPG and vascular calcification intensity [10], and strong clinical evidence supports the claim that OPG is a predictor and marker of vascular calcification in coronary artery disease, diabetes, and chronic kidney disease [11].

Concerning atherosclerosis, high values of serum/plasma OPG are characteristic of coronary artery disease, unstable angina, elevated systolic blood pressure, diabetes, and peripheral arterial disease [28,29]. Moreover, OPG was correlated with carotid plaque vulnerability [30] and coronary artery calcification severity and progression [31,32]. OPG showed an independent association with left-ventricular hypertrophy in males and left-ventricular function in both sexes [33]; the increases in the myocardial and blood levels were described in HF due to acute ischemic events [34].

Less is known concerning RANKL, with some studies indicating that it is a calcification promoter, but others failing to demonstrate any effect. RANKL-transfected mice with overexpression in the SMCs did not present calcium deposition in the aortic roots [35]. An experimental approach, which used bone-marrow-derived macrophages that were co-cultured with vascular SMCs, demonstrated that macrophages’ RANKL treatment induces IL-6 and TNF-α production, which finally governs the vascular SMC-mediated calcification when these are exposed to a high-phosphate-containing medium [36].

In our study, PWV was significantly correlated with serum OPG and the OPG/RANKL ratio. The association between elevated OPG and PWV is in line with other, similar observations. Serum OPG concentration correlated significantly with cfPWV in hypertensive patients [37]. This relationship has also been confirmed in subclinical atherosclerosis: in osteoporotic postmenopausal women with cardiovascular risk factors but no coronary artery disease, serum OPG proved to be an independent predictor of normal-ranged PWV [38]. Investigating peripheral arterial disease vs. age- and AB0-blood-group-matched controls, it was found that patients with critical limb ischemia and non-0 blood groups possess higher plasma OPG values [29]. In the study of Buleu et al., which was performed on patients with HF and coronary artery disease vs. controls, OPG values were positively correlated with cfPWV. The mean PWV scores in both groups were below 10 m/s [39]. In 120 hemodialysis patients studied by Hou et al., higher tertiles of OPG were associated with greater cfPWV values and lower intact parathyroid hormone levels and were predictors of AS together with age, the presence of diabetes, and high serum calcium [40]. Scialla et al. also confirmed a strong, positive relationship between OPG and AS in 226 chronic kidney disease patients, independently of other confounders, such as GFR, albuminuria, serum calcium and phosphate, the presence of secondary hyperparathyroidism, and traditional cardiovascular risk factors. In this study, the first and second OPG tertiles possessed normal PWV values, and only the highest tertile had elevated PWV [41].

When we categorized our patients into two groups, PWV^lo^ and PWV^hi^, with a cut-off value of 10 m/s, the PWV^hi^ group was older, had a higher ejection fraction, higher serum OPG, a higher OPG/RANKL ratio, and a higher intima-media thickness, along with lower GFR values.

Interestingly, PWV was correlated positively with serum OPG and the OPG/RANKL ratio in the whole and PWV^hi^ group, but not in the PWV^lo^ group. When patients were analyzed according to the EF, OPG, and OPG/RANKL, only cases with EF > 30% showed a positive, significant correlation with PWV. In a multiple linear regression model, serum OPG proved to be a strong predictor of PWV when the model was adjusted for hypertension, left-ventricular end-diastolic diameter, serum sclerostin, and exercise level. Moreover, the highest-to-lowest tertiles of OPG conferred a significant risk with an odds ratio of 3.02 (1.13–8.07) for pathologically increased AS when the model was adjusted for IMT, ejection fraction, and NT-proBNP (first model), and IMT, hypertension, and the presence of clinically overt atherosclerotic manifestations (second model).

In the overall group, we also observed significant correlations between OPG, IMT, and GFR. IMT was a significant predictor of AS, both in the whole group and at the PWV^hi^ subjects in all linear and logistic regression models. Elevated OPG levels in carotid atherosclerosis were previously documented by other authors too. In the study of Aoki et al. [42], high serum OPG was significantly associated with carotid artery calcification but showed no differences in different stages of diabetic nephropathy. OPG has predictive value, even in polyvascular atherosclerotic disease: Morisawa et al. proved that OPG was a predictor of cIMT and early carotid atherosclerosis in coronary disease patients [43]. In the Tromso Study, which was performed on a cohort of 6516 individuals aged 25–85 years, Vik et al. described an age-dependent, divergent relationship of IMT and circulating OPG [44].

In individuals that were <45 years, the risk of having increased IMT in the uppermost tertile of OPG was significantly diminished, while in those that were >55 years, this risk was increased, and the two factors showed a positive correlation [44]. They also found that elderly individuals with echogenic carotid plaques presented lower serum OPG than those with echolucent plaques or the controls [44].

The Wnt/β-catenin signaling promotes the loss of vascular SMC phenotype, promotes osteochondrogenic transdifferentiation, and upregulates RANKL in the vessel wall [12,45]. Thus, canonical Wnt signaling inhibitors, such as sclerostin and DKK-1, are presumably negative regulators of AS; indeed, in several studies, serum sclerostin was significantly increased in chronic kidney disease patients with intensive vascular calcification [12].

Sclerostin is a 22 kDa glycoprotein with anti-anabolic effects and was initially thought to be an osteocyte-specific molecule. It has turned out that its gene, *SOST*, is also expressed in other tissues, such as the lung, bone marrow, heart, and blood vessels. It binds to the LRP5/6 receptors and, in this way, competitively inhibits the canonical Wnt signaling. Serum sclerostin increases in the elderly, where ectopic vascular calcification often occurs [12]. Vascular sclerostin might also drive the calcification paradox: sclerostin-producing transdifferentiated vascular SMCs possibly cause a sclerostin spillover into the circulation, thus contributing to the downregulation of bone formation and promoting osteoporosis [12].

Sclerostin seems to participate in the regulation of both intima and media calcification. In the intima, Wnt signaling is involved in endothelial dysfunction [46], macrophage recruitment, and activation [47], and sclerostin was observed to downregulate the expression of matrix metalloproteinase 9, OPG, and osteopontin genes [48]. Calcification of the vascular media, or the so-called Mönckeberg sclerosis, manifests in concentric calcification of the large, elastic, medium-sized, and small arteries, increasing AS as a direct consequence. In this mechanism, osteochondrogenic transdifferentiation, vascular SMC apoptosis, the loss of calcification inhibitors, and induction of canonical Wnt signaling are characteristic features [12,49]. In a warfarin-treated aorta model, De Mare et al. observed a time-dependent calcification with gradually increasing serum sclerostin levels up to 10 weeks [50]. Aortic SOST mRNA levels were also upregulated [50]. The source of the increased levels of circulating sclerostin could be the transdifferentiated vascular SMCs, and the trigger is possibly the high flux of Wnt/β-catenin signaling.

Circulating sclerostin increases by up to four-fold in chronic kidney disease and hemodialysis patients. However, the exact relationship of sclerostin and vascular calcification is yet to be established since some publications state a negative [51], while others no correlation [52] or a positive correlation between the two [53]. In the study of Chang et al., which was performed on 105 hypertensive patients, serum sclerostin proved to be an independent predictor of AS in a model containing age, intact parathyroid hormone levels, systolic blood pressure, diabetes, and GFR [54]. Sclerostin was different in the low and high AS groups, and the cfPWV values were similar to those measured in our study in both groups. The circulating calcium and phosphorus concentrations were comparable; however, the level of intact PTH and the incidence of diabetes were significantly increased in the high AS group. In the study of Hsu et al., which included 68 renal transplant recipients aged 51 ± 9 years with a GFR of 43.6 ± 22 mL/min, it was found that serum sclerostin, but not DKK-1, was an independent predictor of AS. HF in this cohort was only sporadically present (two cases). AS was measured through brachial–ankle PWV, and OPG correlated significantly with serum sclerostin. The high AS group (15.3 ± 2 m/s for the left-side brachial–ankle PWV and 16.2 ± 2 m/s for the right-side brachial–ankle PWV) showed elevated serum sclerostin, but not increased DKK-1 values [55].

In the research of Wu CF et al., which was performed on 122 hemodialysis and 78 peritoneal dialysis patients, it was found that sclerostin, but not DKK-1 and the mode of dialysis, was an independent predictor of high AS. The median age of patients was 61 years, the median PWV in the high-AS group was 12.3 m/s vs. 7.7 m/s in the low-PWV group, whereas the incidences of hypertension and diabetes were 12.9% and 33%, respectively, and the presence of HF was not evaluated [27].

In our study, serum sclerostin was a significant predictor of AS in the subgroup of PWV < 10 m/s in a multiple linear regression model containing IMT, left-ventricular end-diastolic diameter, and hypertension. Sclerostin showed significant positive correlations with IMT and GFR in the whole study group. However, circulating sclerostin was neither associated with AS across the entire group nor was a predictor of PWV values in the PWV^hi^ subgroup. It is also important to note that the OPG and sclerostin concentrations did not correlate in any of the groups.

There might be some explanations for these results. Despite the normal PWV range, among the 41 patients, 18 showed atherosclerotic manifestations (14 of coronary artery disease, 3 of carotid stenosis, and 2 of peripheral arterial disease). The extent of the real atherosclerotic burden in this group remained unknown, and sclerostin was somewhat lower (231.4 pg/mL vs. 242.6 pg/mL) than in the PWV^hi^ group.

The patient population’s renal function was in the normal range, but the EF was significantly lower (median 30 vs. 40) than in the PWV^hi^ patients. The mean PWV in the PWV^lo^ subgroup was 7.79 ± 0.18 m/s and 11.64 ± 0.20 m/s in the PWV^hi^ group. The diminished EF could determine a downward shift of PWV values, which, in reality, might be closer to the cut-off value. Furthermore, the age was significantly lower, and the incidence of diabetes somewhat, but non-significantly, higher in the PWV^lo^ group. Thus, this group differed in many perspectives from those that were characterized with dominant renal pathologies by the other authors mentioned before.

OPG and sclerostin proved to be predictors of AS in many clinical studies performed on different patient populations. However, according to the literature, it is important to stipulate that OPG is involved in both intima- and media-type calcification, while sclerostin seems to especially regulate the vascular media. The majority of these studies targeted atherosclerotic groups or chronic kidney disease sufferers and did not investigate AS on a background of HF. In non-dialysis chronic kidney disease patients, OPG and sclerostin both proved to be strongly associated with arterial calcification [56]. In our study, OPG was a predictor of AS in the overall group and showed no direct correlation with sclerostin, which was also associated with AS only in patients with normal PWV and in the lowest range. These results suggest that the two biomarkers might characterize different AS stages, possibly due to different calcification patterns.

To our knowledge, this is the first study evaluating the relationship between AS and this combination of biomarkers in a population with HFrEF. Since it is a cross-sectional investigation with a relatively low number of cases, our research suffers from several weaknesses. First, due to the number of cases limitation, it cannot offer high statistical power. Second, we did not use any imagistic method to evaluate the arterial calcification load directly, which would be of interest for comparative analysis.

However, we described a predictive role of OPG for pathologically increased AS and a strong relationship between AS and intima-media thickness in our patient group. These two parameters were proposed as two different but complementary pieces of a holistic picture of arterial dysfunction [6].

## 5. Conclusions

In patients with HFrEF, arterial stiffness is related to established factors of vascular aging and endothelial dysfunction. We found OPG and carotid intima-media thickness to be the most related factors to AS in patients with HFrEF. Serum OPG levels were significantly elevated in subjects with pathologically high AS, whereas sclerostin was not different. In our study, serum sclerostin levels were associated with AS only in patients with normal PWV values. Biomarker correlates of AS generally, and specifically in the setting of HF, may provide prognostic information, and further studies performed on larger patient populations are needed to confirm the role of these two biomarkers in HFrEF with comorbidities.

## Figures and Tables

**Table 1:** Clinical and laboratory variables of the 78 HFrEF patients with low versus high arterial stiffness.

Variables	Median (Quartile Range)/Mean ± SE	PWV^lo^ Group (*n* = 41)	PWV^hi^ Group (*n* = 37)	*p*-Values
Demographic and clinical parameters				
Age (years)	66.5 (58–76)	60 (52–65)	76 (72–80)	<0.0001
Gender (f/m)	21 (26.9)/57 (73.1)	6 (14.6)/35 (85.4)	15 (40.5)/22 (59.5)	0.011
Body mass index (kg/m^2^) *	27.31 ± 0.61	27.32 ± 0.82	27.29 ± 0.92	0.978
Ejection fraction (≤30%/>30%)	43 (57.1)/35 (44.9)	28 (68.3)/13 (31.7)	15 (40.5)/22 (59.5)	0.022
Left-ventricular ejection fraction (%)	30 (25–40)	30 (20–35)	40 (30–40)	0.002
Left-ventricular end-diastolic diameter (mm) *	60.60 ± 1.05	62.76 ± 1.64	58.22 ± 1.18	0.03
Global heart failure (y/n)	57 (73.1)/21 (26.9)	32 (78.1)/9 (21.9)	25 (67.6)/12 (32.4)	0.319
Left ventricular failure (y/n)	44 (56.4)/34 (43.6)	20 (48.8)/21 (51.2)	13 (35.1)/24 (64.9)	0.176
History of atrial fibrillation	18 (23.1)/60 (76.9)	5 (12.2)/36 (87.8)	13 (35.1)/24 (64.9)	0.029
Coronary artery disease (y/n)	33 (42.3)/45 (57.7)	14 (34.2)/27 (65.8)	19 (51.4)/18 (48.6)	0.169
Atherosclerotic disease (y/n)	39 (50)/39 (50)	18 (43.9)/23 (56.1)	21 (56.8)/16 (43.2)	0.364
Hypertension (y/n)	43 (55.1)/35 (44.9)	23 (56.1)/18 (43.9)	25 (67.6)/12 (32.4)	0.043
Diabetes (y/n)	55 (70.5)/23 (29.5)	30 (73.2)/11 (26.8)	25 (67.6)/12 (32.4)	0.626
Peripheral arterial disease (y/n)	7 (9)/71 (91)	2 (4.9)/39 (95.2)	5 (13.5)/32 (86.5)	0.246
Carotid atherosclerotic disease (y/n)	10 (12.8)/68 (87.2)	3 (7.3)/38 (92.7)	7 (18.9)/30 (81.1)	0.178
Valvular disease	31 (39.7)/47 (60.3)	13 (31.7)/28 (68.3)	18 (48.6)/19 (51.4)	0.166
Laboratory parameters and biomarkers				
Hemoglobin (d/dL) *	13.73 ± 0.21	13.97 ± 0.23	13.45 ± 0.36	0.217
Platelet count (k/μL) *	206.75 ± 6.05	205.08 ± 8.27	208.6 ± 9.0	0.774
Glomerular filtration rate (mL/min/1.73 m^2^) *	68.10 ± 2.57	73.07 ± 3.79	62.58 ± 3.22	0.04
C-reactive protein (mg/L)	0.91 (0.25–1.8)	0.68 (0.19–1.72)	1.04 (0.45–2.3)	0.103
Fibrinogen (mg/dL)	359 (295–481)	351 (278–450)	365 (318.3–520)	0.339
Serum iron (μg/dL)	60 (45–84)	65 (46–87)	57 (45–80)	0.612
Glucose (mg/dL)	109 (92–134)	100 (90–115)	119 (95–141)	0.038
Triglicerids (mg/dL)	111.5 (93–150)	110 (96–150)	115 (90–150)	0.96
HDL cholesterol (mg/dL)	45 (36–51)	45 (36.3–55)	44.7 (36–50)	0.591
LDL cholesterol (mg/dL)	96.3 (78.3–125)	96.3 (81.8–120.5)	97 (77–128)	0.72
Uric acid (mg/dL)	6.9 (5.6–8.3)	6.8 (6–8.6)	7 (5.6–8)	0.577
NT pro-BNP (pg/mL)	2089.5 (862.1–3089)	2388 (862.1–3604)	1865 (898.9–2713)	0.309
Osteoprotegerin (ng/mL) *	6.25 ± 0.23	5.77 ± 0.31	6.78 ± 0.31	0.024
RANKL (pg/mL)	138.7 (138.2–148.2)	138.6 (138.2–148.2)	138.7 (138.2–147.6)	0.858
Sclerostin (pg/mL)	239.4 (209.8–360.9)	231.4 (203–288.4)	242.6 (220.3–366.3)	0.201
DKK1 (pg/mL)	304.9 (183.6–583.4)	353.2 (203.6–625.5)	265 (182.8–574.3)	0.251
Osteoprotegerin/RANKL ratio	41.0 (28.9–53.5)	38.3 (26.3–45.3)	50.2 (37.1–56.4)	0.029
Ankle–brachial index	1 (0.87–1.15)	1 (0.9–1.13)	1 (0.87–1.2)	0.705
Intima-media thickness (mm)	1 (0.8–1.3)	0.8 (0.8–1.1)	1.2 (0.9–1.3)	<0.0001
Pulse-wave velocity (m/s) *	9.62 ± 0.26	7.79 ± 0.18	11.64 ± 0.20	<0.0001
Lifestyle and medication				
Exercise level (≤30 min/week/>30 min/week)	43 (57.1)/35 (44.9)	19 (46.3)/22 (53.7)	24 (64.9)/13 (35.1)	0.1
ACE inhibitors (y/n)	33 (42.3)/45 (57.7)	15 (36.6)/26 (63.4)	18 (48.6)/19 (51.4)	0.359
Diuretics (y/n)	67 (85.9)/11 (14.1)	34 (82.9)/7 (17.1)	33 (89.2)/4 (10.8)	0.524
Calcium blockers (y/n)	7 (9)/71 (91)	1 (2.4)/40 (97.6)	6 (16.2)/31 (83.8)	0.048
Angiotensin receptor antagonists (y/n)	6 (7.7)/72 (92.3)	2 (4.9)/39 (95.1)	4 (10.8)/33 (89.2)	0.415
Beta blockers (y/n)	67 (85.9)/11 (14.1)	35 (85.4)/6 (14.6)	32 (86.5)/5 (13.5)	1
Nitrates (y/n)	13 (16.6)/65 (83.3)	5 (12.2)/36 (87.8)	8 (21.6)/29 (78.4)	0.364
Antiaggregants (y/n)	31 (39.7)/47 (60.3)	16 (39)/25 (61)	15 (40.5)/22 (59.6)	1
Anticoagulants (y/n)	49 (62.8)/29 (37.2)	25 (61)/16 (39)	24 (64.9)/13 (35.1)	0.816
Statins (y/n)	30 (38.5)/48 (61.5)	15 (36.6)/26 (63.4)	15 (40.5)/22 (59.5)	0.816

Values of variables with a normal distribution (marked with an asterisk) are represented by the mean ± SE, whereas values of variables with an abnormal distribution are shown as median (quartiles). For the first, the PWV groups were compared using the paired Student’s *t*-test, while for the second, the Mann–Whitney *U* test was used. Values for categorical variables are presented as number (%). y—yes (present), n—no (absent).

**Table 2:** Correlations of PWV in the overall, low, and high arterial stiffness groups.

Variables	All Cases (*n* = 78)		PVW^lo^ Group (*n* = 41)		PWV^hi^ Group (*n* = 37)	
	Spearman/Pearson’s *R*	*p*-Value	Spearman/Pearson’s *R*	*p*-Value	Spearman/Pearson’s *R*	*p*-Value
Age	0.875	<0.0001	0.893	<0.0001	0.571	<0.0001
Body mass index *	−0.123	0.282	0.017	0.918	−0.471	0.003
Left-ventricular ejection fraction	0.344	0.002	0.277	0.079	−0.218	0.195
Left-ventricular end-diastolic diameter *	−0.339	0.002	−0.374	0.016	−0.085	0.616
Hemoglobin *	−0.191	0.094	−0.066	0.679	−0.188	0.263
Platelet count *	0.021	0.850	−0.001	0.483	0.091	0.592
Glomerular filtration rate *	−0.370	0.0010	−0.318	0.043	−0.367	0.025
C-reactive protein	0.259	0.022	0.294	0.054	0.300	0.079
Fibrinogen	0.154	0.177	0.130	0.418	0.096	0.573
Serum iron	−0.141	0.218	−0.041	0.798	−0.332	0.045
Creatinine	0.139	0.226	0.195	0.222	0.285	0.087
Urea	0.189	0.098	0.202	0.206	0.296	0.075
Glucose	0.233	0.040	0.028	0.864	0.126	0.458
Triglicerides	−0.034	0.767	−0.114	0.479	0.027	0.874
HDL cholesterol	0.077	0.504	0.448	0.003	0.003	0.985
LDL cholesterol	−0.101	0.381	−0.032	0.845	−0.246	0.142
Uric acid	−0.087	0.449	−0.012	0.941	−0.122	0.470
NT-proBNP	−0.079	0.491	−0.051	0.751	0.180	0.284
Osteoprotegerin *	0.371	0.0010	0.281	0.074	0.330	0.046
RANKL	−0.042	0.717	−0.169	0.292	−0.053	0.755
Sclerostin	0.291	0.0097	0.371	0.017	0.278	0.095
DKK1	−0.095	0.409	0.175	0.272	−0.109	0.522
Osteoprotegerin/RANKL ratio	0.369	0.0009	0.280	0.077	0.387	0.018
Ankle–brachial index	0.078	0.499	0.046	0.775	0.096	0.574
Intima-media thickness	0.552	<0.0001	0.719	<0.0001	0.068	0.689

In the case of unmarked variables, correlations were calculated using the Spearman rank correlation. For variables marked with an asterisk, correlations were calculated using the Pearson product–moment calculation.

**Table 3:** (**A**) Multivariate linear regression analysis of the factors that were correlated with arterial stiffness (PWV) in the overall patient group (*n* = 78). (**B**) Multivariate linear regression analysis of the factors that were correlated with arterial stiffness in the PWV^lo^ group (*n* = 41).

(A)				
Model 1. Summary of the Stepwise Regression				
Variables	Multiple *R*	*R*-Square Change	F-to-Enter/Rem	*p*-Level
Intima-media thickness	0.500	0.250	25.383	<0.0001
Gender	0.581	0.088	9.920	0.002
Osteoprotegerin	0.629	0.057	6.992	0.010
Hypertension	0.652	0.030	3.762	0.056
Ventricular wall thickness	0.661	0.013	1.634	0.205
Sclerostin	0.671	0.013	1.657	0.202
Exercise score	0.679	0.011	1.426	0.236
(**B**)				
**Model 2. Summary of the Stepwise Regression**				
**Variables**	**Multiple *R***	***R*-Square Change**	**F-to-Enter/Rem**	***p*-Level**
Intima-media thickness	0.675	0.456	32.713	<0.0001
Sclerostin	0.727	0.073	5.884	0.020
Ventricular wall thickness	0.763	0.054	4.749	0.036
Hypertension	0.788	0.039	3.699	0.062

**Table 4:** Multiple logistic regression analysis of the factors that were correlated with arterial stiffness (PWV) in the overall patient group (*n* = 78).

Model 1.		
Variables	Odds Ratio (95% CI)	*p*-Value
Osteoprotegerin (pg/mL), tertile 3: tertile 1	3.02 (1.13–8.07)	0.032
Intima-media thickness (mm), tertile 3: tertile 1	3.75 (1.40–10.01)	0.029
Ejection fraction (>30%/≤30%)	3.16 (1.24–8.00)	0.154
NT-proBNP (low/high)	1.31 (0.50–3.39)	0.920
**Model 2.**		
**Variables**	**Odds Ratio (95% CI)**	***p*-Value**
Osteoprotegerin (pg/mL), tertile 3: tertile 1	3.02 (1.13–8.07)	0.039
Intima-media thickness (mm), tertile 3: tertile 1	3.75 (1.40–10.01)	0.017
Hypertension (yes/no)	2.66 (1.05–6.70)	0.341
Atherosclerotic manifestation (yes/no)	1.67 (0.68–4.11)	0.887

## Data Availability

Data spreadsheet available from E.E.N. (2021), “Stat_data_sOPG and cIMT are Related to High Arterial Stiffness,” Mendeley Data, V1, doi: 10.17632/grttt5ks7h.1.
